# The Evaluation of Physical Activity Habits in North Italian People before and during COVID-19 Quarantine: A Pilot Study

**DOI:** 10.3390/ijerph19031660

**Published:** 2022-01-31

**Authors:** Mario Mauro, Alessia Grigoletto, Maria Cristina Zambon, Marzia Bettocchi, Francesco Pegreffi, Carmela Fimognari, Laura Bragonzoni, Pasqualino Maietta Latessa, Stefania Toselli

**Affiliations:** 1 Department of Life Quality Studies, University of Bologna, 47921 Rimini, Italy; mario.mauro4@unibo.it (M.M.); f.pegreffi@unibo.it (F.P.); carmela.fimognari@unibo.it (C.F.); laura.bragonzoni4@unibo.it (L.B.); 2 Department of Biomedical and Neuromotor Sciences, University of Bologna, 40136 Bologna, Italy; stefania.toselli@unibo.it; 3 Welfare Area and Promotion of Community Wellness, Bologna Municipality, 40126 Bologna, Italy; cristina.zambon@comune.bologna.it (M.C.Z.); marzia.bettocchi@comune.bologna.it (M.B.)

**Keywords:** physical activity, COVID-19 quarantine, health, green spaces

## Abstract

COVID-19 caused a global pandemic state. Many governments enforced quarantines which had several negative effects on peoples’ health. The present study aimed to investigate the social restriction effects on the physical activity (PA) habits of north Italian people and understand whether PA was a healthy support during lockdown. Moreover, it analysed some possible strategies which could promote an active lifestyle when the pandemic ends. A new questionnaire was proposed (Cronbach’s alpha = 0.816), and 309 surveys were collected in people from two Italian regions (53.72% from Emilia-Romagna and 46.28% from Veneto; 62.46% were female and 37.54% were male; and the age range was 46.67 ± 15.45 years). The number of younger people (≤25 years) who practiced PA increased during lockdown (*p* < 0.01); in addition, they were more active than people who were 26–35 years old (*p* < 0.001). The training frequency before COVID-19 was higher in females than males (*p* = 0.01), and the frequency of weekly PA increased during lockdown in groups aged 26–35 years (*p* < 0.001). Despite the fact that PA was a psychological support during lockdown (*p* < 0.001), performing forced home-based PA demotivated people (*p* < 0.001). Finally, people thought to practice outdoor PA (OPA) at the end of lockdown because they wanted to retain contact with nature, which can improve psychological well-being. Future strategies to promote OPA may increase participation in PA, especially in older people.

## 1. Introduction

The emergence of COVID-19 was first observed when cases of unexplained pneumonia were noted in the city of Wuhan, China [1]. The earliest recognized cases of COVID-19 in Wuhan were thought to have occurred in early December 2019. In March 2020, the COVID-19 infection caused a global pandemic state. In December 2021, more than 5.26 billion deaths were registered all over the world, of which more than 134,000 deaths occurred in Italy [2,3]. To contain the virus diffusion, many governments enforced quarantine and isolation measures. During quarantine, which lasted from 8th March to 11th May 2020, the Italian Government prohibited outdoor and social activities, resulting in a reduction of outside (park, gym, sports centre, etc.) physical activity (OPA) and exercise [4]. Several authors published exercise guidelines to help people who were forced to perform home-based PA due to pandemic restrictions [5,6]. Nevertheless, recent studies reported that COVID-19 confinement negatively affected PA habits, decreasing the percentage of people who practiced PA, as well as negatively affecting training parameters, such as frequency, intensity, and volume [7,8,9]. For example, some researchers [10] reported that only 40% of Brazilian adults sampled performed PA during social isolation due to pandemic alert. Moreover, other authors found, through longitudinal research, that a sedentary lifestyle increased during the pandemic period in college students [11]. In contrast with these results, two studies showed increments in time spent practicing PA during lockdown in Canadian [12] and Spanish university students [13]. Therefore, it is unclear whether the COVID-19 confinement has changed population PA habits. Conversely, it is clear that quarantine had negative short- and long-term effects on lifestyles, nutritional habits, body health, and mental health [3]. In addition, a systematic review showed an increased prevalence of psychiatric morbidity and psychological distress, such as anxiety, confusion, sleep disorders, stress, and a depressed mood [14].

The World Health Organization [15] recommended to stay active and perform daily PA in order to prevent the occurrence of health disorders such as hypertension and cardio-vascular, respiratory, and metabolic diseases, as well as to reduce the risk of frailty, sarcopenia, and dementia, especially in older people. In addition, some researchers suggested that the practice of PA strengthens the immune system, causing benefits in the response to viral communicable diseases [16]. A review showed that exercise is an effective treatment for depression in the elderly, acting as a protective factor against neurological disorders, improving the quality of life and mood statuses in patient with Alzheimer’s and Parkinson’s diseases, reducing the perceived fatigue, increasing balance and strength, and positively affecting activities of daily life (ADL), preventing fall risks [17].

To the authors’ knowledge, no study analysed the variation in PA habits in Italian people during the three-month lockdown (8 March to 11 May 2020). Moreover, no investigations were conducted on how people thought to change their PA habits after social restrictions were eased, and which measures could be adopted to promote an active lifestyle at the end of quarantine. So, the aims of this research were:

1. To investigate the physical activity habits of people who lived in two northern regions of Italy (Emilia-Romagna or Veneto) before the COVID-19 and during the first lockdown;
2. To understand whether forced indoor PA, due to COVID-19 emergency restrictions, affected PA habits, and if PA acted in a health support role during quarantine. In addition, we wanted to evaluate whether PA mitigated psychological difficulties;
3. To investigate peoples’ intentions and motivations to practice PA after lockdown.

## 2. Materials and Methods

### 2.1. Study Design

The current study is a cross-sectional design, which used a questionnaire to record and gather data. The surveys were shared on the Internet from 10 May up to 20 May 2020. During this period, the Italian Government declared the end of the first lockdown and many social restrictions were abolished. Italian people could practice activities in outdoor spaces such as parks and/or beaches, and some activities in indoor spaces, respecting emergency measures (wearing a mask, social distancing of almost one meter, body-temperature measurement, and hand sanitation).

### 2.2. Questionnaire

We administered the questionnaire to investigate the participants’ habits before and during the first lockdown (COVID-19) in two Italy regions (Emilia-Romagna, Veneto).

We created the questionnaire with Google Forms and shared it on two global social networks (Facebook^®^, Meta Platforms, Inc., Cambridge, MA, USA; LinkedIn^®^, Microsoft, Sunnyvale, CA, USA). All participants were informed and gave us privacy consent to treat their personal data. They could fill out the survey with no Google sign-in request. They could manually enter all general information or allow social networks to report them. The questionnaire was self-administered in the Italian language. Each completed survey was saved on a Google database, and we gathered all data as an Excel spreadsheet (Microsoft Office^®^, Microsoft Corporation, Redmond, WA, USA).

The questionnaire was of three parts, for a total of 33 questions (Appendix A):

Part one: general and demographic information about participants, such as age (in years), gender, country, education, marital status, number of roommate(s), and the distance (in metres) between their houses and the nearest park (Table 1).

Part two: information about physical activity (PA) habits, with particular regard to outdoor physical activity (OPA) practiced before the social restrictions (9 March 2020) due to COVID-19 (21 questions). The questions included OPA habit information, such as the amount of training per week, minutes of PA per week, type of exercises practised, problems met in doing OPA, and preferences between outdoor and indoor environments, socialization aspects, feelings and sensations related to OPA, self-perceived health, physical conditioning, psychological well-being, and satisfaction in practicing OPA.

Part three: information about PA habits during the first lockdown (12 questions), such as the amount of training per week, minutes of PA per week, type of exercises practised, training session assistance (fitness app, online coach, online friend, social network), difference between PA done before social restrictions, and self-perceptions of PA during lockdown.

The question values were different, and many types of data were collected. Five questions were closed questions that were collected as discrete data scored from 1 (disagreement) to 5 (agreement); seven were closed questions firstly collected as categorical data and then transformed to discrete data; two were closed questions collected as discrete data ranging from 30 to 180 (with a scale of 30), eight items were ‘’true or false” questions collected as binary data; and 11 were open questions collected as categorical data with no transformations. We did not consider 17 questions as items of any scale but as independent questions (questions before COVID-19: 1, 2, 3, 7, 9, 11, 15–21; questions during lockdown: 1, 2, 5, 7). Therefore, we considered 16 questions, in total, as measures of a scale, with a total of 30 items.

The reliability of the questionnaire items was tested through homogeneity and internal consistency. To identify the dimensions of our questionnaire, the exploratory factor analysis (EFA) of tetrachoric correlations was assessed [18]. Due to data heterogeneity, each variable in the scale was transformed into a discrete item, such as a Likert-type scale. To measure the sampling adequacy, the Kaiser–Meyer–Olkin (KMO) value was calculated; values > 0.80 were considered meritorious. To test the null hypothesis, that variables were not intercorrelated, Bartlett’s test of sphericity was performed, and the determinant of matrix correlation value, χ^2^, degrees of freedom (df), and the *p*-value (*p*) were reported. The choice of the number of factors was based on the eigenvalues, and we used the unweighted least-squares method and the Kaiser rule to extract only factors with an eigenvalue ≥1. Finally, the orthogonal Varimax rotation was used and the related χ^2^ and *p*-values (*p*) were settled. Items with a loading value < 0.35 were dropped, and the final model included only items with loadings of ≥0.35 on their specific factors.

Then, to provide a measure of the internal consistency of our questionnaire, we calculated the Cronbach’s alpha on all items and on each factor, respectively. We considered the alpha value as acceptable, ranging from 0.70 to 0.95 [19], and we reported the average interitem correlation and alpha values (α).

### 2.3. Sample Inclusion Criteria

Participant inclusion criteria were an adult age (≥18 years old), being based in Emilia-Romagna or Veneto, and those who were Italian and spoke the Italian language.

### 2.4. Statistical Analyses

We reported the frequencies of occurrence and their percentages (%) for categorical or discrete data and mean ± standard deviation (SD) and the minimum and maximum observed values for numerical (continuous) data. Frequency-point differences between countries, gender, age, and PA habits were analysed by logistic regression and the χ^2^ Statistics were reported. When significant values were found, a post-hoc analysis with a *Z*-test was assessed to look for each group difference, and the Bonferroni correction method was applied to avoid significance bias (α = 0.05/k, where k is the number of group comparisons). The statistic test and *p*-values (*p*) were reported. The within-group analysis was assessed with McNemar’s test, whereas the general mean differences in proportion between PA before COVID-19 social restrictions and during the first lockdown were assessed by the *Z*-test of proportion or the Student’s *t*-test (one-sample or two-sample) and were reported as means (with a 95% confidence interval) and *p*. The one-way ANOVA was carried out to assess PA- related group comparisons. The one-sample *t*-test was used to compare observed mean values with a hypothetical value.

A correlation matrix was carried out to look for linear relationships between many variables. Then, a regression model was carried out to analyse the reasons why participants thought to practice outdoor physical activity after lockdown. The stepwise backward procedure was executed, and only regressors which explain at least 7% of the dependent variable, with *p* ≤ α = 0.05, were included in the model. The goodness-of-fit was reported as an adjusted-R^2^ value and the ANOVA table was presented.

We selected a priori hypothesis test significance levels as equal to 0.05 (α). All statistical analyses were performed by STATA^®^ software (version 17, Publisher: StataCorp. 2021. Stata Statistical Software: Release 17. College Station, TX, USA, StataCorp LP).

## 3. Results

### 3.1. Sample

**Figure 1:**
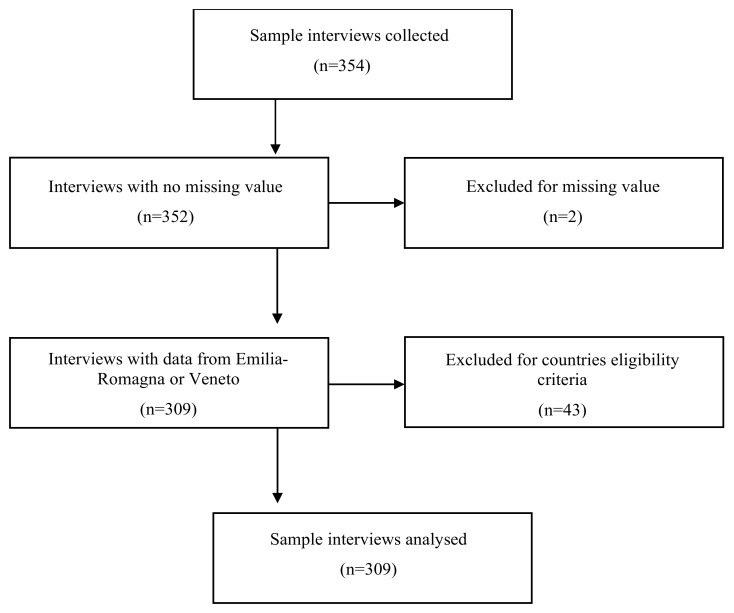
Sample flowchart.

Figure 1 shows the sample’s flow diagram. At the beginning, we collected 354 participant interviews. Of these, two were excluded for missing data and 43 were excluded because participants did not live in Emilia-Romagna or Veneto. Finally, we analysed interviews of 309 subjects.

### 3.2. Participants’ Characteristics

Table 1 shows participant characteristics. Of the participants, 62.46% were women. Each participant included in the study was an adult (≥18 years old) and was not older than 86. The average age was 46.67 (±15.45) years old. Of the participants, 53.72% lived in Emilia-Romagna. Approximately half of the participants were married (49.84%) and almost the 35% had a master’s degree. Every participant lived with an average of 2.67 (±1.28) roommates and 568.55 (±391.8) lived near a park.

### 3.3. Questionnaire Characteristics

Totally, we included 22 items in the model (Table 2). At the beginning, physical activity parameters that quantified and qualified the amount of exercise were analysed by 10 (five before the COVID-19 onset and five during lockdown) items, the health perceptions and improvements were analysed by 13 items (nine before the COVID-19 onset and four during lockdown), and the physical activity problems experienced before COVID-19 and during quarantine were analysed by seven items (six before the COVID-19 onset and four during lockdown). Then, we deleted four items due to no Likert scale, and four items due to poor loading values.

**Figure 2:**
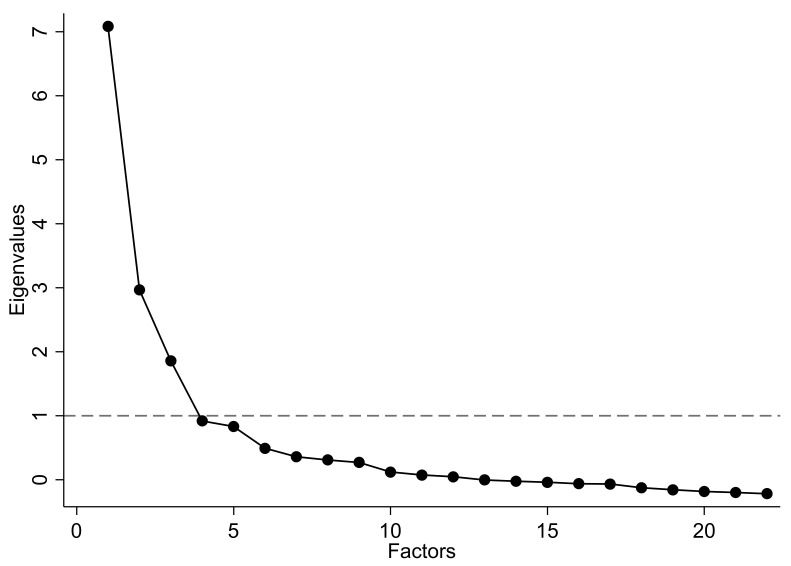
Scree plot of eigenvalues with Kaiser rule (intersecting dash line = 1).

Firstly, we analysed the sampling adequacy (KMO = 0.813), that the correlation matrix determinant equal 0.001, and Bartlett’s test of sphericity (χ^2^ = 2041.082; dfs = 231; *p* < 0.001). Figure 2 shows the scree plot of eigenvalues with the Kaiser rule. Three factors met our criteria and the Varimax rotation reported a LR test of significant results (χ^2^_231_ = 2058.86; *p* < 0.001; *n* = 124). Factor one (health) contained 11 items and interpreted the perceived psychophysical health; factor two (PA parameters) contained seven items and interpreted the PA characteristics which described the participants’ habits in doing exercise; and factor three (PA problems) contained four items and interpreted the perceived problems which could reduce participants’ participation in practicing PA.

Finally, we assessed the Cronbach’s alpha on 22 items. The last row shows all Cronbach’s alpha values. We used the mean test scale on standardized items, deleting missing values from the analysis. The average interitem correlation on 124 observations was 0.168 and the scale reliability coefficient (α) was 0.816. In addition, we reported each consistency factor data: factor one, with an average interitem correlation = 0.556, α = 0.926; factor two, with an average interitem correlation = 0.336, α = 0.78; and factor three, with an average interitem correlation = 0.347, α = 0.69.

### 3.4. Physical Activity Characteristics

We analysed the differences of participants’ PA habits before COVID-19 and during the first lockdown. Table 3 reports the statistical outcomes of the proportions of people who practiced PA before the COVID-19 onset and during lockdown. Generally, we did not find significative differences in the proportion of people who practised PA before COVID-19 and during lockdown (McNemar’s χ^2^ = 0.93; *p* = 0.39). We did not observe significant statistical differences between male and female PA habits before COVID-19 (χ^2^ = 0.0020; *p* = 0.96), during the first lockdown (χ^2^ = 0.266; *p* = 0.61), and within female (McNemar’s χ^2^ = 0.97; *p* = 0.39) and male (McNemar’s χ^2^ = 0.10; *p* = 0.76) subgroups, respectively, before and during the lockdown. No significant statistical differences were found between the proportion of people who practiced PA and lived in Emilia-Romagna or Veneto before COVID-19 (χ^2^ = 2.73; *p* = 0.1) and during the lockdown (χ^2^ = 0.171; *p* = 0.68). Conversely, significant differences were found in the proportion of people who did PA between the pre-COVID-19 and lockdown periods between regions (*Z* = 2.35; *p* = 0.01). No significant differences were found in the percentages of people who practiced PA within Emilia-Romagna (McNemar’s χ^2^ = 0.02; *p* = 0.89) or Veneto (McNemar’s χ^2^ = 1.42; *p* = 0.29) before and during lockdown.

Regarding age, the lowest frequencies of practicing PA during lockdown were observed in groups who were 26–35 years, whereas the highest was observed in the 18–25-year-old group. If we consider the change in physical activity practiced before COVID-19 and during lockdown, a significant increasing trend (within-group comparison) of the frequencies was observed for younger age groups (*Z* = 2.73; *p* < 0.01), while a decreasing trend was shown by subjects in the three older age groups. A significant difference in the proportion of people who practiced PA before the COVID-19 onset was found between the age groups (χ^2^ = 20.33; *p* = 0.001) and the post-hoc analysis showed that there were lower proportions in the group that was 26–35 years old compared to 46–55 years old (*Z* = −3.47; *p* < 0.001) and the group that was 56–65 years old (*Z* = −3.9; *p* < 0.001). Moreover, significant differences in proportions were found between age groups during lockdown (χ^2^ = 16.48; *p* < 0.01), specifically between people who were 18–25 and 26–35 years old (*Z* = 4.06; *p* < 0.001), and between people who were 26–35 and 46–55 years old (*Z* = −2.8; *p* = 0.002). Finally, many significant statistical outcomes were found in the proportion of differences pre-lockdown and during lockdown between groups who were 18–25, 46–55, 56–65, and over 66, respectively (*Z* = 4, *p* < 0.001; *Z* = 10.7, *p* < 0.001; *Z* = 5.11, *p* < 0.001), groups who were 26–35 and 56–65 years old (*Z* = 4.12; *p* < 0.001), and groups who were 36–45 and 56–65 years old (*Z* = 6.6; *p* < 0.001).

**Figure 3:**
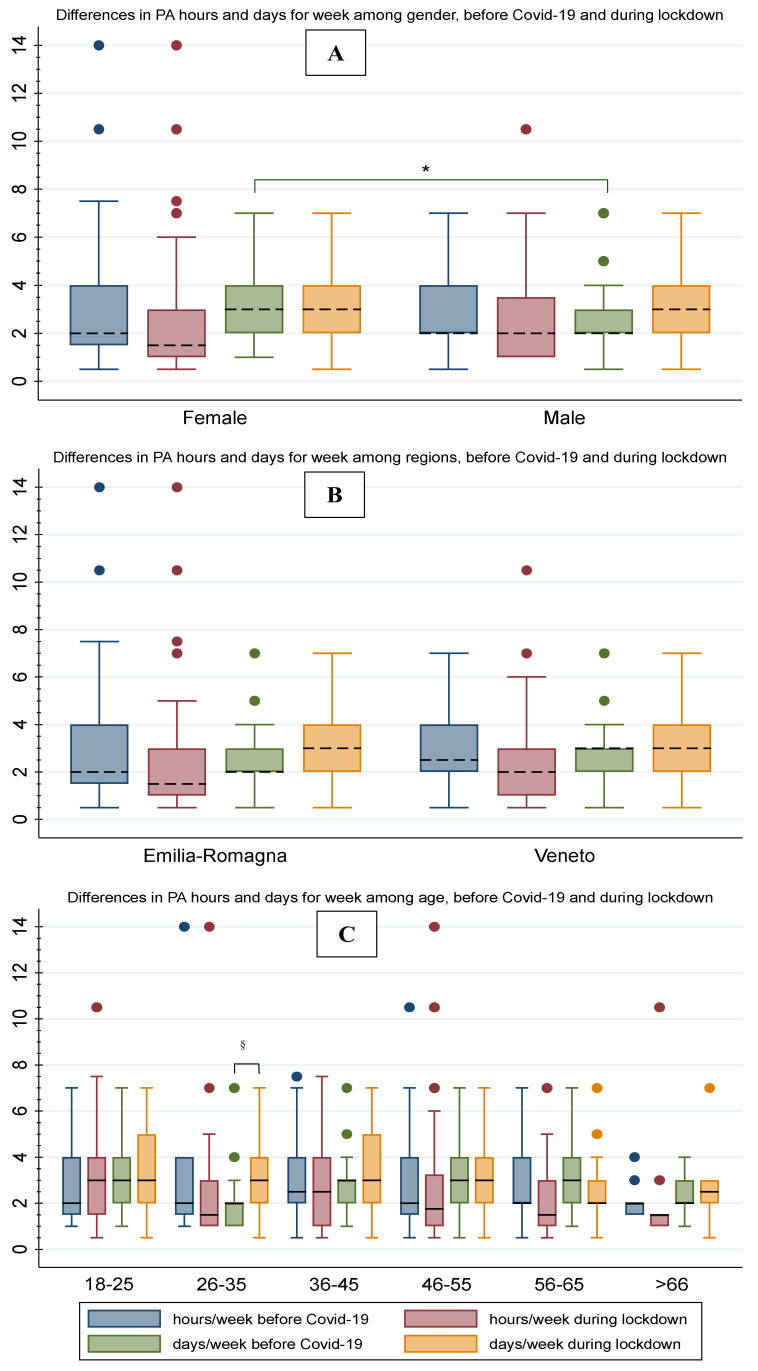
Different distributions among hours and days per week of physical activity practiced before and during COVID-19 social restrictions, comparing gender (**A**), region (**B**), and aging classes (**C**). Note: *, *p* ≤ 0.01; §, *p* ≤ 0.05.

Figure 3A shows differences in hours and days per week of PA as a function of gender; Figure 3B shows the differences in hours and days per week of PA practised before COVID-19 and during lockdown among people who lived in Emilia-Romagna and Veneto; and Figure 3C shows differences in hours and days per week of PA among age groups. Table 4 shows related statistical outcomes. Generally, we did not find significant differences (t_162_ = 1.19; *p* = 0.24; C.I.: −0.12–0.48) among hours per week before COVID-19 (2.81 ± 1.79, C.I.: 2.53–3.1) and during lockdown (2.63 ± 2.45, C.I.: 2.25–3.01). Conversely, we found a significant increase (t_170_ = −1.922; *p* = 0.05; C.I.: −0.56–0.007) in the days per week of practice before COVID-19 (3.05 ± 1.855, C.I.: 2.76–3.32) and during the first lockdown (3.32 ± 1.86, C.I.: 3.05–3.60). We did not observe significant differences in hours per week of PA before COVID-19 among regions (F_1, 202_ = 0.08; *p* = 0.78), gender (F_1, 202_ = 1.55; *p* = 0.21), and age (F_5, 198_ = 0.62; *p* = 0.69), and during lockdown among regions (F_1, 246_ = 0.21; *p* = 0.64), gender (F_1, 246_ = 0.59; *p* = 0.44), and age (F_5, 242_ = 1.25; *p* = 0.29).

We did not find differences in days per week of PA before COVID-19 among regions (F_1, 204_ = 1.73, *p* = 0.19) and age (F_5, 200_ = 1.76; *p* = 0.123), and in days per week of PA during lockdown among gender (F_1, 254_ = 0.02; *p* = 0.88), regions (F_1, 253_ = 0.12; *p* = 0.73), and age (F_5, 250_ = 0.65; *p* = 0.66). Conversely, we found a significant statistical difference in days per week of PA before COVID-19 in gender, where a higher frequency was observed in females (F_1, 204_ = 6.26; *p* = 0.0132). In addition, we found a significant difference in days per week of PA practice before COVID-19 (2.25 ± 1.80, C.I.: 1.4003.09) and during the lockdown (3.55 ± 1.57; C.I.: 2.81–4.28) in the age group of 26–35 years, where the frequency increased during lockdown (paired *t*-test_19_ = −4.64; *p* < 0.001; C.I.: −1.89–−0.71).

We found different percentages of home-based training types practiced during lockdown: the most of participants did walking (27.80%), followed by well-being PA (19.69%), resistance exercises (15.44%), and combined PA (8.88%).

We also analysed whether practising PA with no training group negatively affected the participants’ motivation, comparing the observed mean result (0.66 ± 0.47, C.I.: 0.59–0.73) with a hypothetical value of a neutral result (H_0_: mean = 0.5) and a significant difference was detected (t_189_ = 4.75; *p* < 0.001). The same method was carried out to evaluate whether PA acted as a physical and psychological support during lockdown (mean = 0.75 ± 0.43. C.I.: 0.70–0.80) and a significant difference was found (t_303_ = 10.23; *p* < 0.001). In addition, participants’ answers to the question about the reasons why PA was supportive, and “psychological support” was the most common response (44.55%).

Finally, we investigated whether, and why, people who practised forced at-home PA for pandemic restrictions wanted to practice outdoor PA when pandemic social restrictions ended. In order to assess a regression model, at the beginning we analysed 11 variables (Table 5); of these, only two regressors (psychological well-being, missing nature contact) met our criteria and explained 54.6% (adj. R^2^) of the dependent variable. Table 6 shows the regression model outcomes and which variables could explain the variability of the peoples’ decision to do OPA after lockdown.

## 4. Discussion

The first purpose of our study was to investigate how physical activity habits in people who lived in Emilia-Romagna or Veneto changed due to pandemic social restrictions. We observed a significantly different trend between people form Emilia-Romagna and Veneto, which shows that people who lived in Veneto became more active during lockdown. Despite other authors [9] showing decreased levels of PA during the pandemic social restrictions (from 69% to 39%), we found several significant differences in frequencies of people who practiced PA between the period before and during the first lockdown, where the proportion of younger people who were 18–45 years old increased their PA during lockdown, rather than the decreasing their percentage of PA, such as people who were more than 46. However, the authors looked for percentage differences in people who were classified as very active (performing at least 30 min of vigorous activity five times per week), whereas our purpose was to investigate the general population PA habits. Moreover, different pandemic rules were adopted by Brazilian and Italian governments.

When compared to the Italian sample, our results disagree with outcomes reported from research in which the authors found decreased level of PA in undergraduate students [8]. Our study showed significant increments, both in the proportion of people who did PA, and in the hours per week spent doing PA in the 18–25-year-old group during lockdown. However, the authors included, in their questionnaire, only one question related to PA habits, which participants could answer alone as to whether their PA decreased, increased, or did not change during lockdown.

Conversely, older age negatively affected the PA habits, because people who were 46 or older and practiced PA before COVID-19 reduced it during the first lockdown. According to some authors [20] the reduction in PA observed during quarantine is a serious concern for older adults, as they are typically less active compared to younger people and are more prone to chronic diseases. In addition, our results showed that participants who were 66 or more practiced PA for fewer hours per week than younger people and they also showed the lowest frequency of training sessions (days per week). However, we found the percentage of the frequency of PA of almost 40%, which was higher than that observed by Ammar et al. [7,21,22] who found lower frequencies of PA in three different studies during lockdown (22.7%, 24%, and 35%). In our sample, only people who were 56–65 years old showed a frequency of PA lower than 35%. We think that the lower frequency of PA in older people is caused by the lack of a kinesiologic specialist who usually, in these age groups, helps the subjects during the training session, improving the participant’s mood state, and providing safety. In addition, the socialisation factor, as an activity group, and the missed contact with nature could have negatively affected the desire of performing exercise.

When we analysed gender habits in PA frequency, we found a significant difference before COVID-19, since females showed a higher number of days per week of exercise than males. Regarding this difference, our hypothesis is that physiological sex characteristics, such as hormonal status, affect acute and chronic responses post-exercise, which led female subjects to perform workouts with lower intensities that requires less rest times and allows them to maintain a higher training frequency [23]. Moreover, our result is in accordance with the results of previous studies which found gender differences in exercise motivation, where men preferred performing sports for competitive reasons, whereas women are more inclined to do well-being exercises as yoga and pilates, or to home-based free-weight exercise [24,25,26].

To understand PA habits during the pandemic emergency, we also asked to participants what kind of home-based exercise they practiced. Our results showed higher percentage in walking activities, followed by well-being PA (yoga, pilates, and postural gymnastic) as suggested by the WHO [27]. We think the rationale of this is, in fact, that walking activities do not need gym equipment and coach/trainer monitoring [28]. However, we did not investigate whether participants who practiced walking activities used the treadmill or any other equipment.

Despite the fact that not all the people in our sample changed PA habits during quarantine and, for the most part, maintained an active lifestyle, our suggestion is to perform daily exercise to enhance health, especially in older people whose habits were most affected during quarantine [6]. We think that more care is needed for older people, because they are more easily exposed to several diseases caused by inactive lifestyle habits and PA seems to be the better strategy to avoid them. Daily campaigns to promote and diffuse exercise benefits may slow down aging processes, preventing many pathologies.

The second purpose of this research was to investigate whether PA was a psycho-physical and mental support during lockdown, and whether PA mitigated the psychological difficulties that arose during quarantine. In a systematic review, it was reported that quarantine negatively affected mental health, causing hostility, anxiety, stress, depression, and altered sleep quality [14]. In the current study, we found that PA acted as a support for people during quarantine, especially as a psychological support. In addition, PA positively affected motivation, which may play a key role in reducing sedentary lifestyles [29]. According with these results, some researchers [10] found that people who did PA during lockdown exhibited lower stress levels and better sleep levels than people who did not practice PA. Moreover, two authors [12] found that participants who were more physically active showed greater mental health scores, whereas inactive participants who became more active during lockdown reported lower levels of anxiety. We believe the rationale of this is in the fact physical activity produces benefits on mental health and the endocrinal system, positively affecting peoples’ lives [27].

The last goal of the current research was to investigate whether people who practiced outdoor physical activity before the COVID-19 emergency missed it, and whether they wanted to practice physical activities in an outdoor environment at the end of the pandemic restrictions. We wanted to investigate this because before the COVID-19 quarantine, more than the 82% of active people sampled by our survey preferred to perform physical activity in blue spaces such as parks or beaches than the indoor environment. Our results showed that the most relevant reasons for which people wanted to perform OPA after social restrictions were missing contact with nature and the psychological impact of outdoor exercise. To our knowledge, no researchers investigated factors related to OPA characteristics, and no comparisons are possible. In line with these outcomes, we think that the future promotion of OPA in a natural environment, after the pandemic global status, may involve a great part of the north Italian population, improving PA habits promoting health.

### Limitations

One limitation of this study is the small sample size. Other questionnaires were proposed during this period which were met greater responses, collecting bigger samples [26,30,31].

The second limit is that we analysed only two Italian regions and we cannot extend our results to the whole Italian population.

In addition, we produced a new questionnaire which makes it hard to compare our results to other studies outcomes. Moreover, the pre-pandemic physical activity was measured retrospectively and no cross-sectional observation at a specific time was assessed. The retrospective outcomes could be affected by participant perceptions, and this could be the reason why no differences in the time spent practicing PA before and during quarantine was observed. Likewise, we did not ask participants information about PA parameters such as intensity and volume, because it is not easy to calculate them without a trained specialist. However, our purpose was to evaluate the general characteristics of PA and changes in population habits.

## 5. Conclusions

Physical activity is a good strategy to prevent health disorders during quarantine and social restrictions. It improves peoples’ mood and maintains peoples’ active lifestyles. However, aging could negatively affect the involvement in daily PA, and older people decreased their time spent practicing PA, increasing the risk of falls and the onset of several diseases. Moreover, home-based exercise seems to badly impact adult motivation. Future strategies to promote outdoor activities in natural environments may increase adult participation in PA and positively affect their habits.

## Figures and Tables

**Table 1:** Participant characteristics.

	Category	Frequency	Percent (%)	*n*
Gender	Female	193	62.46	309
	Male	116	37.54	
Country	Emilia-Romagna	166	53.72	309
	Veneto	143	46.28	
Marital status	Married	154	49.84	309
	Single	86	37.83	
	Engaged	57	18.44	
	Widow	10	3.24	
	Not declared	2	0.65	
Education	Master’s degree	108	34.95	309
	Diploma	106	34.3	
	Bachelor	36	11.65	
	Ph.D.	27	8.74	
	Primary or secondary school	26	8.41	
	Other	6	1.94	
	Mean (±std)	Min	Max	n
Age (year)	46.67 (±15.45)	18	86	309
Roommate number	2.67 (±1.28)	0	6	309
Distance between house and park (metres)	568.55 (±391.8)	0	4000	248

**Table 2:** Rotated factor loadings (pattern matrix) and Cronbach’s alpha values.

	Factor 1	Factor 2	Factor 3
Items (22)	Health (11)	PA parameters (7)	PA problems (4)
Psychological well-being	0.94	−0.04	0.04
Perceived mood	0.93	0.03	0.02
General well-being	0.92	0.07	−0.04
Physical well-being	0.91	−0.01	0.02
Stress reduction	0.9	−0.08	0.07
Self-gratification	0.88	0.01	0.05
Anxiety reduction	0.87	−0.06	0.05
Outdoor PA in future	0.66	0.01	0.08
Missing nature aspects	0.54	0.05	0.2
Perceived fatigue	0.51	−0.07	0.18
Missing social aspects	0.39	−0.2	0.19
PA day/week before COVID-19	−0.12	0.78	0.13
PA day/week lockdown	0.03	0.75	−0.01
PA hour/week before COVID-19	0.04	0.74	0.14
PA hour/week lockdown	0.09	0.73	−0.08
PA day/week goal after lockdown	−0.16	0.56	−0.06
Importance of group for PA	0.04	−0.4	0.18
Self-perceived PA condition	−0.17	0.38	0.18
Group level	0.07	0.12	0.78
Group coordination	0.12	0.05	0.72
Group participant necessity	0.03	0.03	0.6
Transport to PA place	−0.02	−0.15	0.48
α = 0.816	α = 926	α = 0.78	α = 0.69

Note: Loadings ≥ 0.38 are shaded.

**Table 3:** Differences in proportion of people who practiced PA.

Characteristic		Pre	Lock	Between Groups						Within-Groups		*n*
				Pre		Lock		∆				
				χ^2^	*P*	χ^2^	*P*	*Z*	*P*	*χ^2^* or *Z*	*P*	
General		0.81	0.84							0.93	0.39	309
											
Gender	M	0.81	0.83	0.002	0.96	0.266	0.61	1.04	0.15	0.97	0.39	116
	F	0.81	0.85							0.1	0.76	193
												
Region	E	0.84	0.85	2.73	0.1	0.17	0.68	2.35	0.01 *	0.1	0.89	166
	V	0.77	0.83							0.68	0.29	143
												
Age	18–25	0.77	0.94	20.33	0.001 *	16.48	<0.01 *	Z_18–25 vs. 46–55_ = 4	<0.001 *	2.73	<0.01 *	66
	26–35	0.63	0.67	Z_26–35 vs. 46–55_ = −3.47	<0.001 *	Z_18–25 vs. 26–35_ = 4.06	<0.001 *	Z_18–25 vs. 56–65_ = 10.7	<0.001 *	0.41	0.68	51
	36–45	0.78	0.84					Z_18–25 vs. >66_ = 5.11	<0.001 *	0.77	0.443	49
	46–55	0.89	0.88	Z_26–35 vs. 56–65_ = −3.9	<0.001 *	Z_26–35 vs. 46–55_ = −2.8	0.002 *	Z_26–35 vs. 56–65_ = 4.12	<0.001 *	0.24	0.807	82
	56–65	0.93	0.85					Z_36–45 vs. 56–65_ = 6.6	<0.001 *	1.34	0.18	46
	> 66	0.87	0.8							0.49	0.624	15

Note: E, Emilia-Romagna; F, female; Lock, lockdown; M, male; n, number of observations; Pre, before COVID-19; V, Veneto; Z, Statistic Z; χ^2^, chi-squared value; *, statistical significance.

**Table 4:** Mean differences in PA days and hours per week.

		Pre COVID-19		Lockdown		∆	*t*	*p*
General	Hours/Week	2.81 ± 1.79 (2.53; 3.1)		2.63 ± 2.45 (2.25, 3.01)		(−0.12; 0.48)	1.19	0.24
	Days/Week	3.05 ± 1.85 (2.76; 3.3)		3.32 ± 1.86 (3.05; 3.60)		(−0.56; 0.007)	1.92	0.05
		*F*	*p*	*F*	*p*			
Region	Hours/week	0.08	0.78	0.21	0.64			
	Days/week	1.73	0.19	0.12	0.73			
Gender	Hours/week	1.55	0.21	0.59	0.44			
	Days/week	0.02	0.88	6.26	0.01 *			
Age ^†^	Hours/week	0.62	0.69	1.25	0.29			
	Days/week	1.76	0.123	0.65	0.66			

Note: ∆, differences between pre COVID-19 and lockdown; *, statistical significance; †, participants who were 25–36 showed a significant increment in days/week during lockdown (∆ C.I. = 0.71–1.89; t19 = −4.64; *p* < 0.001).

**Table 5:** Correlation Matrix.

	OPA	Nature	Group	Physical	Psychol	Anxiety	Stress	Gratific	Mood	Fatigue	General	Motivation
OPA	1											
Nature	0.6571	1										
Group	0.2499	0.247	1									
Physical	0.5567	0.4641	0.2233	1								
Psychol	0.5942	0.4889	0.2738	0.8827	1							
Anxiety	0.5326	0.4761	0.2165	0.731	0.7608	1						
Stress	0.5277	0.4723	0.2059	0.7678	0.8147	0.9102	1					
Gratific	0.5012	0.3968	0.2404	0.7663	0.7465	0.7009	0.7453	1				
Mood	0.5465	0.4335	0.2619	0.7896	0.8145	0.8197	0.8492	0.828	1			
Fatigue	0.226	0.179	0.2131	0.3024	0.3295	0.3415	0.3241	0.3291	0.3673	1		
General	0.5471	0.4625	0.2702	0.8148	0.7848	0.6976	0.7487	0.7812	0.8069	0.3039	1	
Motivation	0.0565	0.0894	0.2192	0.0843	0.0533	0.1178	0.1263	0.0694	0.1102	0.0679	0.0262	1

Note: OPA, outdoor physical activity after COVID-19; Nature, missing nature contact during lockdown; Group, missing training group during lockdown; Physical, physical well-being OPA-related; Psychol, psychological well-being OPA-related; Anxiety, anxiety reduction OPA-related; Stress, stress reduction OPA-related; Gratific, self-gratification OPA-related; Mood, mood improvement OPA-related; Fatigue, fatigue reduction OPA-related; General, general well-being OPA-related; Motivation, alone at-home-based PA on motivation.

**Table 6:** Regression model for Outdoor PA after COVID-19 variability.

Source	SS	df	MS	*F*	*p*	n
Model	281.72	2	140.858	175.67	<0.001	291
Residual	230.93	288	0.80184			
Total	512.65	290	1.76774			
R^2^ = 0.549		adjusted R^2^ = 0.546		root MSE = 0.895		

	Coeff.	S.E.	t	*p*	95% C.I.	
Intercept	0.195	0.224	0.87	0.385	−0.246	0.64
Pshychol	0.453	0.06	7.62	<0.001	0.336	0.57
Nature	0.494	0.044	11.21	<0.001	0.41	0.58

Note. SS, sum of squares; df, degrees of freedom; MS, mean of squares; S.E., standard error.

## Appendix A

General Information

First name: ……… Last name: ………

Birth date: ……… Birth place: ………

Gender: ………

Marital status: ………

Education level: ………

Profession: ………

Number of people in your house: ………

Green spaces in your house (balcony/garden/terrace): ………

Park near house (yes/no): ……… if yes, distance: ………

District of residence: ………

Information about Physical Activity before COVID-19

1. Did you practice outdoor physical activity?
2. If no, why?
3. How often?
  □ Once per week
  □ Two times per week
  □ Three times per week
  □ Four times per week
  □ Five times per week
  □ Everyday
  Less than one time per week
4. How many minutes?
  □ 30
  □ 60
  □ 90
  □ 120
  □ 150
  □ 180
  □ More than 180
5. You think your weekly physical activity is:
  □ More than enough
  □ Enough
  □ Not enough
  □ Poor
  □ I do not know
6. What kind of outdoor physical activity did you practice?
  □ Walking
  □ Running
  □ Resistance training (bodyweight, free-weight, gym equipment)
  □ Well-being (yoga, pilates, postural gymnastic)
  □ Functional training
  □ Other (please specify)
7. How long have you practiced PA?
8. What is the major problem in practicing outdoor physical activity? (Score 1 to 5, where 1 is absolutely disagree and 5 is absolutely agree)
  - Transport
  - Inconsistency
  - No group coordination
  - Conflicted group
  - People with compromised autonomy in the group
  - Inadequate space
9. Did you practice PA alone?
10. If not, how important is group activity for you? (Score 1 to 5)
11. If you practiced outdoor PA, why did you prefer it to indoor PA?
12. After you have practiced outdoor physical activity, what are your feelings? (Score 1 to 5)
  - Physical well-being
  - Psychological well-being
  - Stress reduction
  - Anxiety reduction
  - Self-satisfaction
  - Mood improvement
  - Fatigue
  - General well-being
13. What did you think about your physical conditioning? (Score 1 to 5)
14. What did you think about your psychological well-being? (Score 1 to 5)
15. Do you think outdoor PA improves your health?
16. Why?
17. Does outdoor PA satisfy you?
18. Did you perform OPA during winter?
19. If yes, with the same activity and frequency?
20. If no, did you perform indoor PA during winter?
21. If yes, what kind of activity?

Information about Physical Activity during first lockdown

1. Did you practice PA?
2. What kind of PA did you practice?
  □ Walking
  □ Running
  □ Resistance training (bodyweight, free-weight, gym equipment)
  □ Well-being (yoga, pilates, postural gymnastic)
  □ Functional training
  □ Other (please specify)
3. How often?
  □ Once per week
  □ Two times per week
  □ Three times per week
  □ Four times per week
  □ Five times per week
  □ Everyday
  □ Less than one time per week
4. How many minutes?
  □ 30
  □ 60
  □ 90
  □ 120
  □ 150
  □ 180
  □ More than 180
5. What about modality?
  □ Autonomously
  □ With a friend
  □ With an online trainer
  □ With a fitness app
  □ With some social network
6. If you practiced group activity, did activity by yourself impact your mood?
  □ A lot
  □ Quite
  □ No
  □ A little bit
7. Was physical activity a support during this period? If yes, why?

Evaluate the following answers (Score 1 to 5, where 1 is absolutely disagree and 5 is absolutely agree)

1. I missed contact with nature
2. I missed group socialization
3. I thought I would practice outdoor physical activity when lockdown ends
4. Do you think you will practice outdoor PA again?
5. How often?
